# Moonlighting Proteins and Their Role in the Control of Signaling Microenvironments, as Exemplified by cGMP and Phytosulfokine Receptor 1 (PSKR1)

**DOI:** 10.3389/fpls.2018.00415

**Published:** 2018-03-28

**Authors:** Helen R. Irving, David M. Cahill, Chris Gehring

**Affiliations:** ^1^Monash Institute of Pharmaceutical Sciences, Monash University, Melbourne, VIC, Australia; ^2^La Trobe Institute for Molecular Science, La Trobe University, Bendigo, VIC, Australia; ^3^Faculty of Science Engineering and Built Environment, Deakin University, Geelong, VIC, Australia; ^4^Department of Chemistry, Biology and Biotechnology, University of Perugia, Perugia, Italy

**Keywords:** calcium, cyclic nucleotides, cyclic GMP (cGMP), kinases, intracellular signals, microenvironment, molecular crowding, phytosulfokine receptor (PSKR1)

## Abstract

Signal generating and processing complexes and changes in concentrations of messenger molecules such as calcium ions and cyclic nucleotides develop gradients that have critical roles in relaying messages within cells. Cytoplasmic contents are densely packed, and in plant cells this is compounded by the restricted cytoplasmic space. To function in such crowded spaces, scaffold proteins have evolved to keep key enzymes in the correct place to ensure ordered spatial and temporal and stimulus-specific message generation. Hence, throughout the cytoplasm there are gradients of messenger molecules that influence signaling processes. However, it is only recently becoming apparent that specific complexes involving receptor molecules can generate multiple signal gradients and enriched microenvironments around the cytoplasmic domains of the receptor that regulate downstream signaling. Such gradients or signal circuits can involve moonlighting proteins, so called because they can enable fine-tune signal cascades via cryptic additional functions that are just being defined. This perspective focuses on how enigmatic activity of moonlighting proteins potentially contributes to regional intracellular microenvironments. For instance, the proteins associated with moonlighting proteins that generate cyclic nucleotides may be regulated by cyclic nucleotide binding directly or indirectly. In this perspective, we discuss how generation of cyclic nucleotide-enriched microenvironments can promote and regulate signaling events. As an example, we use the phytosulfokine receptor (PSKR1), discuss the function of its domains and their mutual interactions and argue that this complex architecture and function enhances tuning of signals in microenvironments.

## Introduction

**FIGURE 1:**
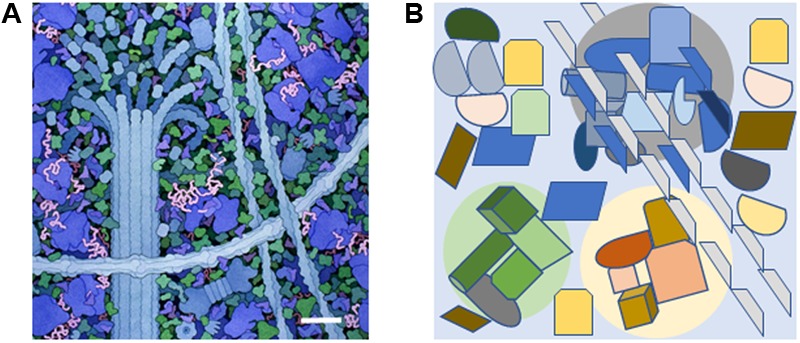
The molecularly crowded cytoplasm and the presence of small signal-enriched microenvironments within the cytoplasm. **(A)** A small portion of the cytoplasm depicting molecular crowding as illustrated by David S. Goodsell, the Scripps Research Institute. Cytoskeletal components are shown along with ribosomes (large blue molecules). The scale bar = 30 nm and is based on the measured width of ribosomes (Haga et al., 1970). **(B)** Diagram showing how specific signal-enriched areas can occur in the cytoplasm. Three areas with different micro-enriched signals (gray, pale green, and pale yellow) are depicted that affect separate groups of proteins and represent areas with diameters of 100–200 nm. The microenvironments may be created by proteins within the group or generated from external sources such as ion fluxes from internal organelles or the extracellular environment.

Often, despite the beautiful illustrations from Goodsell (1993, 2016), it is forgotten how crowded it is within the cytoplasmic space (Fulton, 1982). The term molecular crowding is used to indicate that 25 to 40% of space in the cytoplasm is occupied by many different, large biomolecules (**Figure 1A**). The number of individual biomolecules is estimated to range from 10 to 1000 molecules per cell (Srere, 1967; Luby-Phelps, 2000; Ellis, 2001). Water is closely associated with the surface of large biomolecules while the flow apart from this encasing layer reflects diffusion (Srere, 1980, 1981, 2000; Saenger, 1987; Luby-Phelps, 2000, 2013; Ellis, 2001; Saxton, 2012; Phillip and Schreiber, 2013).

In plant cells, the cytoplasm is found in a relatively narrow layer between the plasma membrane and large organelles such as the vacuole, compounding the problem of molecular crowding. Underlying the plasma membrane is a network of cytoskeletal proteins that support and interact with other proteins, and are involved in organelle movement (Williamson, 1993; Shimmen, 2007; Goldstein and van de Meert, 2015). Cytoplasmic streaming involves various biophysical pathways resulting in movement that can be along cellular edges or alternatively create turbulence throughout the cytoplasm (Goldstein and van de Meert, 2015). Over short distances diffusion of small molecules is faster than cytoplasmic streaming (Vestergaard et al., 2012). However, if the small molecules have affinity to large biomolecules, they may have restricted capacity to diffuse (Geremia et al., 2006).

Molecular crowding occurs despite the limited copies of individual proteins present (Luby-Phelps, 2000, 2013; Ellis, 2001), so correct spatial arrangements of individual enzymes is necessary for signals to be relayed through signal networks to elicit cellular responses. Several metabolic pathways or metabolons employ molecular channeling to efficiently deliver the product from the first enzyme to form the substrate for the next enzyme (Srere, 1985, 2000; Miles et al., 1999; Winkel, 2004; Moller, 2010; Sweetlove and Fernie, 2013; Zhang et al., 2017). Correct positioning of individual enzymes and scaffold proteins enhances signal cascades via molecular channeling (Rohwer et al., 1998; Wheeldon et al., 2016). Positioning in this way creates subcellular microenvironments containing micro-cues of concentrated signaling molecules that in turn activate downstream points of signal cascades, thereby emphasizing the importance of spatial and temporal regulation of protein expression.

The crowded intracellular space combined with cellular compartmentalization and intracellular molecular gradients, have led biological systems toward microenvironments (or localized signaling circuits). We propose that a component of these signaling circuits are moonlighting proteins. In a general sense, moonlighting proteins are proteins that can perform more than one function and/or act in more than one spot in the cell (Jeffery, 2003, 2009, 2014). Examples of the latter type of moonlighting proteins include various mitochondrial proteins that also act in the nucleus (Monaghan and Whitmarsh, 2015) where they contribute to cellular signaling pathways. It is thought that during evolution, ancestral proteins have acquired additional functions including transcriptional regulation and signal transduction. For instance, it has been suggested that many of the newly emerging RNA-binding proteins are moonlighting as they have enzymatic activities, for example in metabolism, as well as functioning as regulators of transcription and RNA turnover (Hentze and Preiss, 2010; Marondedze et al., 2016b). In this perspective, we will concentrate on moonlighting proteins with roles in signal transduction. Many of these moonlighting proteins are receptor kinases that contain a cytosolic main function, a kinase, and an additional cryptic function, a cyclase (Wong and Gehring, 2013; Wong et al., 2015). The spatial arrangement of these two domains is somewhat unexpected and since both enzymatic activities are affected by second messengers such as calcium ions and the catalytic product of each domain, these moonlighting proteins are likely to serve as molecular tuners. For these reasons, we propose that such moonlighting signaling proteins are well-suited to operate in or generate subcellular microenvironments containing ions or small molecules that provide points of control in signal cascades (**Figure 1B**). Concepts of metabolons and molecular crowding are well-established while the concept of proteins generating their own small molecule microenvironmental regulatory milieu is less established. This perspective focuses on recent advances in cGMP signaling in plants, and how enigmatic activity of moonlighting proteins can contribute to regional intracellular microenvironments. First, we discuss the importance of small incremental changes in cGMP microenvironments and then we use the phytosulfokine receptor (PSKR1) as an example of a moonlighting protein that generates phospho- and cGMP-microenvinvironments.

## Signal Strength and Specificity

A considerable body of literature exists on the biological functions and mechanisms of action of cyclic nucleotide signaling in lower and higher eukaryotes (Lemtiri-Chlieh et al., 2011). In fact, cGMP and cAMP are accepted as key signaling molecules in developmental and environmental stress response cascades. However, acceptance that cyclic nucleotides have such a role in plant signaling was slow. One reason was that, in plants, cellular cyclic nucleotide levels seem generally lower than in animals or lower eukaryotes (Newton et al., 1999; Gehring, 2010; Marondedze et al., 2017). An additional reason for the initially reluctant acceptance of these signaling molecules in higher plants was that molecular evidence for mononucleotide cyclases in higher plants only came after the first plant draft genome was published in 2000 (Ludidi and Gehring, 2003). Since the publication of the first mononucleotide cyclase, the number of identified and experimentally tested mononucleotide cyclases has increased steadily and there are indications that there are >50 candidate cyclases in the *Arabidopsis thaliana* proteome and that they come in many different domain organizations (Meier et al., 2007; Wong and Gehring, 2013). The multitude of candidates and domain partners points to a diverse spectrum of biological functions for mononucleotide cyclases and their catalytic products.

Invariably the question of how a single messenger, like cAMP or cGMP, is capable of triggering highly specific responses to different developmental and/or environmental stimuli arises. It seems obvious that saturating the cell with either cAMP or cGMP cannot be the answer. To illustrate the point, such an approach would be like attempting to regulate traffic flow in a city with only one “gigantic traffic light” that is either red or green. Since the “gigantic traffic light” is unlikely to work, two solutions come to mind. One solution relies on strict compartmentalization of the messenger(s) and the other on the combination and integration of several messengers, e.g., cAMP/cGMP with cytoplasmic calcium ions and/or pH. A recent review has highlighted the interplay of calcium ion signatures with cGMP in plant–microbe interactions (Yuan et al., 2017). Specific response signatures and cooperation between messengers arises through spatial clustering of stimulus-dependent cyclases and their downstream signaling components and/or through the specific binding of the cyclic nucleotides to effector molecules such as kinases (Kwezi et al., 2011; Isner and Maathuis, 2016; Wheeler et al., 2017) or channel subunits (Hoshi, 1995; Zelman et al., 2012). A recent study using a constitutively expressed mammalian guanylate cyclase in Arabidopsis that produced intracellular cGMP levels >50-fold above normal resulted in mis-signaling and down-regulation of many proteins in systemic acquired resistance (Hussain et al., 2016). This study and others where calcium ion influxes flood intracellular compartments (Sanders et al., 2002; Yuan et al., 2017) highlight the need for transient and controlled levels of signaling molecules to generate appropriate responses to environmental and developmental stimuli within defined cytoplasmic areas or cellular compartments.

An affinity pull-down approach has been applied to obtain a cGMP-dependent interactome (Donaldson and Meier, 2013; Donaldson et al., 2016) where several of the cGMP-binding candidates have critical functions in the Calvin–Benson–Bassham cycle and the photorespiration pathway and they also contain cyclic nucleotide-binding domains. It is conceivable that the enzyme activity of these molecules may be directly or indirectly modified by cGMP. Since the Calvin–Benson–Bassham cycle is confined to the stroma of the chloroplast, we might imagine cGMP is generated specifically in the stroma to modulate these enzymes without affecting, for example, cGMP-dependent channels found in the plasma membrane of guard cells. Incidentally, it has also been demonstrated that the activity of the cGMP-binding photorespiration enzyme glycolate oxidase (GOX1) is dampened by cGMP and NO treatment. Since GOX1 produces H_2_O_2_ in response to *Pseudomonas* (Pst DC3000 AvrRpm1), it implicates cGMP-mediated processes in the cross-talk between NO and H_2_O_2_ signaling during defense responses (Donaldson et al., 2016).

If we agree that the “gigantic traffic light” does not work, we may find it easier to accept that small transients in cellular cAMP and cGMP are not a problem, but rather the solution to highly differentiated stimulus-specific cellular signaling in plants. The “gigantic traffic light” has additional implications; predominantly in relation to systems-based investigations of cAMP- and cGMP-dependent processes where the experimental set-up includes cell-permeant cyclic mononucleotides at high concentrations. Such investigations, particularly at the system level, can give insights into cyclic mononucleotide-dependent phosphoproteome (Marondedze et al., 2016a), but merely identify target rather than resolve stimulus-specific signaling cascades. In addition to the generators of the cyclic nucleotide signal, we must also consider the signal-off state. The role and, to this date, lack of genetic evidence for suitable phosphodiesterases that degrade cyclic nucleotides to mononucleotide phosphates has been excellently reviewed (Grosse and Durner, 2016). To generate greater insights into the formation of subcellular microenvironments, specific signaling pathways need to be examined in detail as complex interactions are likely between proteins and the immediate microenvironment (**Figure 1B**). Below we describe the evidence supporting the formation of a subcellular microenvironment surrounding the moonlighting phytosulfokine receptor (PSKR1) as an example of how plants may utilize enigmatic enzymatic centers in homeostatic function.

## PSKR1 and the Formation of Microenvironments

Phytosulfokine (PSK) was first discovered as a secreted sulfated pentapeptide promoting growth in cell cultures and the receptor via ligand-based affinity chromatography (Matsubayashi and Sakagami, 1996; Matsubayashi et al., 2002). Characterization of PSK:PSKR1 ligand-receptor interactions has shown that they have extensive roles in plant growth and development (Wheeler and Irving, 2010; Matsubayashi, 2014; Sauter, 2015). PSKR1 is a member of the leucine-rich repeat receptor like kinase family that typically contain a large extracellular ligand-binding domain composed of leucine-rich repeats, a single transmembrane spanning domain and an intracellular catalytic kinase domain (Matsubayashi et al., 2002). There are five genes encoding PSK that are expressed in different tissues of the plant (Matsubayashi et al., 2006). Active PSK needs to be sulfated and this is achieved by tyrosylprotein sulfotransferase (TPST) found in the Golgi apparatus. Genetic approaches have been a powerful tool used to study PSK:PSKR interactions in plants and much of this work has been carried out in Arabidopsis. All sulfated residues are removed in *tpst* mutants as TPST is the single enzyme catalyzing sulfation of tyrosine residues in Arabidopsis, while triple knockouts of *pskr1, pskr2* and *pysr1* are used to create the null *pskr* receptor background. These plants show defects in growth and development. Specifically, the *pskr* null background has reduced root and shoot growth and revealed that the PSK:PSKR receptor system is involved in promoting root and shoot growth in addition to roles in development of xylem vessels and pollen tubes (Sauter, 2015). Analysis of plant pathogen interactions has revealed that PSKR also has roles in protecting plants. While plant growth is promoted by PSK, pattern-triggered immune responses such as those activated by the biotrophic bacteria *Pseudomonas syringae* are attenuated by PSK (Igarashi et al., 2012). Interestingly, although the *pskr* or *tpst* null mutants are more resistant to biotrophic pathogens such as *P. syringae*, the oomycete *Hyaloperonospora arabidopsidis* and the nematode *Meloidogyne incognita*, they are more susceptible to necrotrophic pathogens such as the fungus *Alternaria brassicicola* (Mosher et al., 2013; Rodiuc et al., 2016). One of the features biotrophic pathogens have in common is that on penetrations of host cells, they stimulate the formation of specialized cell structures (e.g., haustoria) and it appears that these pathogens have co-opted the PSK signaling system to promote cell differentiation (Rodiuc et al., 2016). Moreover, PSK is expressed in nodules in *Lotus japonicus* and application of exogenous PSK increases nodule numbers (Wang C. et al., 2015) where rhizobia may be co-opting PSK in the formation of the specialized nodule.

**FIGURE 2:**
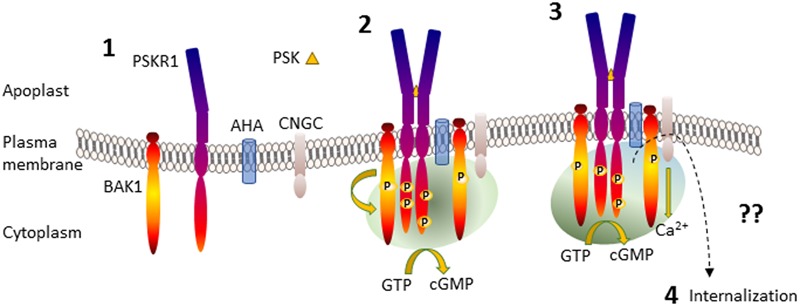
Generation of PSKR1 activated subcellular cGMP enriched-microenvironment. Part 1 shows the anchoring of the proteins in the plasma membrane. Part 2 shows PSK binding to the ligand-binding domain of PSKR1 and receptor dimerization with BAK1 and phosphorylation, formation of an association with AHA and CNGC and the generation of a microenvironment containing cGMP generated via PSKR1 (green shading) although other sources are possible. Part 3 depicts the activation of CNGC and development of an additional calcium ion microenvironment surrounding the receptor complex (blue shading) that enhances cGMP production, which in turn inhibits kinase activity. Part 4 is not depicted and represents receptor deactivation possibly by endocytosis resulting in internalization as indicated by a dashed arrow. For further details see the text. AHA, H^+^-ATPases; BAK1, BRI1 associated kinase; CNGC, cyclic nucleotide-gated channel; PSK, phytosulfokine; PSKR1, phytosulfokine receptor.

Binding of PSK to PSKR1 stimulates allosteric changes throughout the receptor resulting in heterodimerization interactions with other integral membrane proteins such as the somatic embryogenesis receptor-like kinase (SERKs) including BRI1-associated receptor kinase (BAK1)/SERK3 (Wang J. et al., 2015). Leucine-rich repeat receptor like kinase homo- and heterodimerization is well-established following the characterization of the brassinosteroid receptor brassinosteroid insensitive 1 (BRI1) (Clouse, 2011). Similarly, the damage ligand, AtPep1, binds to its leucine-rich repeat receptor like kinase, PEPR1, and causes heterodimerization with BAK1 (Tang et al., 2015). BAK1 is a promiscuous molecule that was first discovered associated with BRI1 but also interacts with many other LRR RLKs (Chinchilla et al., 2009). Life time fluorescence imaging revealed that PSKR1 interacted with H^+^-ATPases AHA1 and AHA2 and also BAK1 to form a receptor complex (Ladwig et al., 2015). This complex involving PSKR1, BAK1, AHA1, and AHA2 also associates with the cyclic nucleotide-gated cation channel 17 (CNGC17) although PSKR1 does not directly bind to it (Ladwig et al., 2015). Since CNGC17 is regulated by calmodulin and cGMP (Zelman et al., 2012; Fischer et al., 2013, 2017), there is a possibility that a localized receptor complex microenvironment involving calcium ions and possibly cyclic nucleotides such as cGMP is generated (**Figure 2**).

*In vitro* studies provided the first clues that PSKR1 may form subcellular microenvironments containing cGMP. Within the kinase domain of PSKR1 is a sequence motif predictive of a guanylate cyclase center (Kwezi et al., 2007). Studies using recombinant cytoplasmic domains of PSKR1 revealed that the protein could indeed produce cGMP albeit at low levels that were enhanced with additional calcium ions (Kwezi et al., 2011; Muleya et al., 2014, 2016). Both BRI1 and PEPR1 also have a similar guanylate cyclase center that can generate cGMP (Kwezi et al., 2007; Qi et al., 2010; Wheeler et al., 2017). Initial studies used a small recombinant fragment of the BRI1 kinase domain containing the guanylate cyclase center that showed cGMP generation (Kwezi et al., 2007) although a later study using most of the kinase domain failed to show cGMP production (Bojar et al., 2014). However, a more sensitive detection method has since demonstrated cGMP production by the full length BRI1 kinase domain (Wheeler et al., 2017). Notably, this recombinant protein also contained the N and C terminal regions necessary for homodimerization (Bojar et al., 2014) that has been predicted to be necessary for the catalytic conversion of GTP to cGMP (Freihat et al., 2014; Muleya et al., 2016). Arabidopsis mesophyll protoplasts treated with exogenous application of PSK generated increased cGMP levels compared to the controls treated with the non-sulfated backbone PSK pentapeptide (Kwezi et al., 2011). Furthermore, transfection of protoplasts with full-length PSKR1 considerably raised basal levels of cGMP (Kwezi et al., 2011). In addition, the ligand for PEPR1, Pep1, stimulates intracellular increases in cGMP measured using an *in vivo* cGMP reporter in root cells (Ma et al., 2012). Within the catalytic guanylate cyclase center, G924 in PSKR1 is predicted to have roles in determining substrate specificity for GTP (Sunahara et al., 1998; Tucker et al., 1998; Wong and Gehring, 2013; Wong et al., 2015). When glycine is mutated to lysine (G924K), cGMP production is reduced *in vitro* (Kwezi et al., 2011; Muleya et al., 2014). Complementation studies with this full length mutant in the *pskr* null background showed that it could not restore root growth (Ladwig et al., 2015). However, although the G924K mutant did not significantly impair phosphorylation of the SOX substrate (Muleya et al., 2014), it was unable to phosphorylate myelin basic protein (Kaufmann et al., 2017), so questions arise about its ability to properly phosphorylate PSKR1 downstream substrates *in vivo*.

Phosphorylation has long been recognized as a means of regulation of proteins as the number and specific residues phosphorylated create ionic enriched micro-environments. Like BRI1, PSKR1 is a dual kinase with the ability to phosphorylate tyrosine as well as threonine and serine residues (Oh et al., 2009; Muleya et al., 2016) and has a complex autophosphorylation profile involving dimerization (Hartmann et al., 2015; Muleya et al., 2016). Complementation studies with the PSKR1 kinase inactive mutant K762E in the *pskr* null background demonstrated that kinase activity is essential for root and shoot growth (Hartmann et al., 2014). The phosphorylation status of PSKR1 is important in regulating the guanylate cyclase activity as well as its kinase activity. For instance, phosphorylated mimetic mutations of phosphorylated serine residues located at the juxtamembrane region enhance kinase activity *in vitro* and also have differential effects on growth, promoting growth in the root but not the shoot (Muleya et al., 2016; Kaufmann et al., 2017). Interestingly, the mimetic non-phosphorylated mutation decreases kinase activity and both mutations are associated with a lack of guanylate cyclase activity, whereas other mutations that modify kinase activity (Y888E or Y888F) have little effect on guanylate cyclase activity (Muleya et al., 2016). These differences may be related to the ability of the phospho-mimetic mutants to form homodimers, which are important at least in the tyrosine kinase activity and potentially in the guanylate cyclase activity (Muleya et al., 2016).

There appears to be considerable intramolecular crosstalk occurring as not only is kinase activity associated with specific residues being available for phosphorylation, but it is decreased by the guanylate cyclase product cGMP. Thus PSKR1 can generate an enriched environment of cGMP that in turn suppresses its predominant kinase function. The PSKR1 receptor complex also includes CNGC17 (Ladwig et al., 2015) which can be regulated by cGMP. Together these findings suggest that cGMP acts as a regional traffic signal within the PSKR1 receptor complex (**Figure 2**). BRI1 also displays a complex autophosphorylation status that impacts on its effects on growth (Clouse, 2011). Like PSKR1, cGMP inhibits kinase activity in BRI1 and certain kinase inactive mutants no longer generate any cGMP (Kwezi et al., 2011; Wheeler et al., 2017). To date, it is not known how cGMP inhibits kinase activity of PSKR1 or BRI1 but it is potentially by binding at intracellular allosteric sites on the receptors. Following activation of PSKR1 and PePR1 and association with BAK1, they are then internalized by a clathrin-dependent pathway that is important in sustaining immune responses (Mbengue et al., 2016; Rodiuc et al., 2016).

Intracellular calcium ion concentration is tightly controlled to ensure that cells can rapidly respond to specific patterns of spatial and temporal changes in calcium ion levels (Ehrhardt et al., 1996; Sanders et al., 2002; Kudla et al., 2010; Steinhorst and Kudla, 2013; Chen et al., 2015; Yuan et al., 2017). Changes in calcium ion concentration begin at localized points in the cell via influx from external sources that in turn can amplify release via both internal and further external sources generating calcium ion waves (Tuteja and Mahajan, 2007). Calcium ion concentration is returned to basal levels via various internalization mechanisms. *In vitro* studies have shown that PSKR1 can directly respond to physiological calcium ion concentrations of 0.1–10 μM via a reversible inhibition of kinase activity (Muleya et al., 2014). Notably at these same concentrations, guanylate cyclase activity is enhanced and this appears to be a reciprocal effect as lower calcium ion concentrations are associated with high kinase activity (Muleya et al., 2014). It is possible that even higher levels of calcium ions override this effect since 100 μM did not inhibit kinase activity using myelin basic protein as a substrate (Kaufmann et al., 2017). PSKR1 also contains a predicted calmodulin binding site within its kinase domain that interacts with calmodulins (Hartmann et al., 2014). Although complementation studies using PSKR1 W831S mutants in the *pskr* null background suggest that calmodulin binding is necessary for growth responses (Hartmann et al., 2014) it has since been identified that this mutation removes kinase activity (Kaufmann et al., 2017). There is a need to investigate if specific PSK signaling modulates changes in calcium ion concentrations and how these may affect the receptor and immediate surrounding microenvironment.

## Conclusion and Future Questions

We argue that small amplitude signals have a critical part to play in plant homeostasis and that these begin with the development of micro-signaling environments within the cytoplasm that set up the potential for specific signal cascades. Such cascades are likely to exhibit skewed subcellular distribution of the moonlighting proteins and gradients of their small molecule products. Growing pollen tubes in fact have marked distribution gradients in calcium ions that are independent of cytoplasmic streaming and diffusion (Tuteja and Mahajan, 2007). Advances in spatial and concentration level detection methods will enable demonstration of the skewed distribution of the moonlighting proteins and their products which are restricted to small defined areas possibly due to affinity with other molecules that prevents their diffusion.

We have focused on how PSK signals via the PSKR1 receptor to generate a complex series of cross-talk situations on PSKR1 itself involving phosphorylation, cGMP and calcium ions that also influence other proteins such as CNGC17 present in the receptor complex (**Figure 2**). Ligand activated PSKR1 raises cGMP levels that in turn activate CNGC17 increasing calcium ion influx. The consequences of activating this moonlighting protein system are twofold. Firstly, increases in calcium ion concentration potentiate cGMP production amplifying the signal. Secondly, increases in calcium reduce kinase activity of PSKR1 (and its downstream signal cascades). However, there may be increases in activity of other kinases that are dependent on cGMP and/or calcium ion and/or calmodulin. If this is the case, then the moonlighting action of PSKR1 would be a tuner switch for two or more distinct kinase dependent cascades. How changes in calcium ions and cGMP modulate PSK signaling is not clear and will be subject of future investigation. An area that is particularly worth focusing on is how PSK signaling is modulated and switched from growth promotion, to specialized cell development and/or defense responses and the role of subcellular microenvironments in these pathways. In conjunction with these questions, we need to consider the role of phosphodiesterase and suppressors of other signaling molecules that contribute to changes in cellular microenvironments.

We predict that changes at the intracellular microenvironmental level are likely to affect more than homeostasis of individual proteins and will actually have an important part in initiating cellular signaling pathways to maintain plant function in response to rapidly changing environmental conditions and stresses. Understanding the roles of cellular microenvironments is a current focus in diverse research areas as it is now evident that the location of ribosomes influences the mRNA that will be translated and the post-translation modifications that follow (Shi et al., 2017; Simsek et al., 2017).

## Author Contributions

HI and CG conceived the perspective and drafted the manuscript. All authors revised the manuscript, agreed to content, and approved the final version.

## Conflict of Interest Statement

The authors declare that the research was conducted in the absence of any commercial or financial relationships that could be construed as a potential conflict of interest.
